# P-1288. Delineating the impact of New Delhi Metallo-β-lactamases towards in vitro susceptibility among carbapenem-resistant Acinetobacter baumannii clinical isolates using zinc limited media

**DOI:** 10.1093/ofid/ofaf695.1476

**Published:** 2026-01-11

**Authors:** Saipriya Gadiraju, Alissa Padgett, Andrew J Fratoni, Tomefa E Asempa, David P Nicolau

**Affiliations:** Hartford Hospital, Hartford, CT; Hartford Hospital, Hartford, CT; Hartford Hospital, Hartford, CT; Hartford Hospital, Hartford, CT; Hartford Hospital, Hartford, CT

## Abstract

**Background:**

Previous studies have shown that antimicrobial susceptibility testing in zinc-limited broth (developed to mimic physiologic conditions) better predicts *in vivo* efficacy among MBL producing Enterobacterales and *Pseudomonas aeruginosa*. Our objective was to extend this assessment to *Acinetobacter baumannii* (AB) and evaluate the *in vitro* activity of sulbactam-durlobactam (SUD), meropenem (MEM), and aztreonam (ATM) against NDM producing AB isolates in zinc limited media.

**Table 1:**
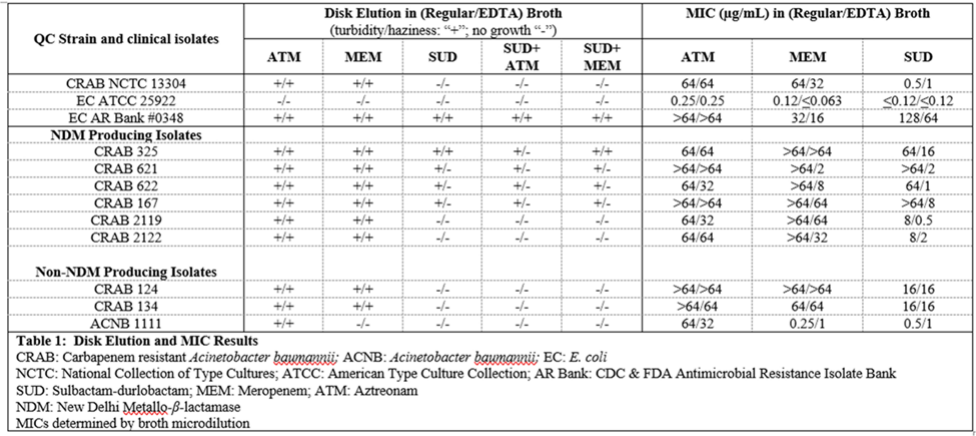
Disk Elution and MIC Results CRAB: Carbapenem resistant Acinetobacter baumannii; ACNB: Acinetobacter baumannii; EC: E. coli NCTC: National Collection of Type Cultures; ATCC: American Type Culture Collection; AR Bank: CDC & FDA Antimicrobial Resistance Isolate Bank SUD: Sulbactam-durlobactam; MEM: Meropenem; ATM: Aztreonam NDM: New Delhi Metallo-β-lactamase MICs determined by broth microdilution

**Methods:**

Nine clinical AB isolates (6 NDMs) and 3 QC isolates were evaluated (Table 1). Zinc limited media was utilized for all experiments using CAMHB supplemented with 30 mg/L of EDTA. *In vitro* activity was assessed via broth microdilution (BMD) MICs. Potential for synergy was evaluated via broth disk elution (DE) with the following drugs and combinations: no disk (growth control), ATM, MEM, 2xSUD (2 disks), ATM + 2xSUD, and MEM + 2xSUD. Disks of SUD 10/10 µg, ATM 30 µg, and MEM 10 µg were added to 5 mL broth tubes to achieve breakpoint relevant concentrations of 4/4, 6, and 2 µg/mL, respectively. Each tube was assessed for growth (not susceptible) or no growth (susceptible). Results were compared with identical experiments using standard CAMHB without EDTA.

**Results:**

QC strains and clinical isolates without NDM demonstrated MIC and DE agreement with and without EDTA as expected. NDM isolates were non-susceptible by BMD to SUD in regular broth but became susceptible/intermediate with EDTA in all except 1 isolate (CRAB 325). MEM MICs were unchanged in EDTA broth relative to standard broth in 3 of 6 NDM-harboring isolates, likely due to presence of other resistance mechanisms (eg. OXA-23). The supplementation of EDTA led to inhibition with SUD alone in 3 of 4 NDM isolates that showed positive growth in regular broth. The addition of ATM to SUD in EDTA broth was the only combination that resulted in inhibition relative to SUD alone (CRAB 325).

**Conclusion:**

The susceptibility profile of isolates without NDM was not altered by limiting zinc. By altering the hydrolytic capacity of NDM through addition of EDTA, CRAB isolates became increasingly susceptible to SUD alone despite harboring an array of alternative resistance mechanisms. Combining SUD + ATM warrants further investigation as a therapeutic strategy for NDM-producing CRAB.

**Disclosures:**

Andrew J. Fratoni, PharmD, Qpex Biopharma, Inc.: Grant/Research Support Tomefa E. Asempa, PharmD, Innoviva: Grant/Research Support David P. Nicolau, PharmD, Carb-X: Grant/Research Support|Innoviva: Advisor/Consultant|Innoviva: Grant/Research Support|Shionogi: Advisor/Consultant|Shionogi: Grant/Research Support

